# Down with the Butcher

**Published:** 1895-12-21

**Authors:** 


### Down with the Butcher.

This is, perhaps, a harsh translation of the opinions expressed in the little volume to which Dr. George Keith gives the modest title "A Plea for a Simpler Life," but, in truth, what he has done is to inaugurate a crusade against over-eating. It is true that we occasionally say in a general way that "more people kill themselves by over-eating than by over-drinking," but there is no general belief that gluttony is as great a vice as drunkenness, and the teetotaller has, even in the vulgar mind, a respect accorded to him which is not shared by the vegetarian. It is true that the glutton does not, when gorged, murder his wife and set fire to his house. His is a soporific aud stertorous weakness, which generally tends to make him feel at peace with all men—at least until indigestion sets in. Yet gluttony saps the foundations of life as surely as drunkenness, and indeed Dr. Keith quotes the opinion of an insurance official who told him that after both had come to a mature age he would consider a better "life," from an actuarial point of view, that of a Highlander who had drunk whisky freely but had lived on plain fare all his days, than that of a "well-fed Yorkshire teetotaller."

Now this, in days when we hear so much of the evils of strong drink, is rather a startling doctrine, but Dr. Keith, from a long medical experience, brings abundant evidence to uphold it. Especially is he averse from the habit of "keeping up the strength" of invalids by giving them too nourishing food. If the body is weak and exhausted, he argues, it is unable to deal with much or strong food, and instead of "keeping up the system," as we fondly think when pressing nourishment on the invalids, we are forcing to unwelcome exertion feeble organs that stand in need of rest. He quotes the bulletins issued concerning one distinguished invalid as alternating between two extremes. One set contained the news that the patient was exhausted, the other that he had revived and was able to take a little nourishment. Dr. Keith's inference is that the collapse was the result of the feeding enforced by anxious nurses, the improvement the result of the exhaustion which made such feeding impossible, and that if, when the patient recovered consciousness, the process of "keeping up his strength" by means of food had been let alone, he would have had a much better chance of recovery.

It is true that the experiments of "fasting men," uncomfortable creatures though they are even to hear about, have proved that the human body can do without food longer than was formerly thought possible outside the region of miracles. It is true also that there are many people who would be none the worse for a moderate dose of starvation, whose organs of digestion would be only too thankful to have a little leisure left them in which to overtake the arrears of work with which they are burdened. It is further true that the too common habit of continually taking drugs in order to eliminate superfluities from the system is, on the face of it, an insult to nature, and is, if we think of it, a habit only a shade less offensive than those methods of renewing a jaded appetite practised by the Roman Emperors. The Church, when it ordained the Friday's fast and the longer Lenten diminution of diet, did well for its worshippers in the old days when feasting and revelry were a matter of course with all who could attain them, and the Protestant substitute of blood-letting was probably not such a rash thing as we of this neurotic age might think. To distended blood-vessels, to gasping hearts, to livers torpid through overwork, let there be rest and a diminution of labour. There are many invalids in all classes, going from one pill to another, from one doctor to another, from one mineral spring to another, according to their fancy or their purse, who would get well just as quickly if they saved their money, stayed at home, and starved; but there are others with whom such treatment would hardly succeed. The diseases of the poor are not often due to surfeit, and, in spite of Dr. Keith, we think that the administration of nourishment is often an essential part of the cure.

As a matter of fact, Dr. Keith's book deals chiefly with persons of a plethoric habit and their diseases. Often these diseases are so subtle that only a skilled eye would call them such; the casual observer only notes that So-and-So is getting stout, but, seeing his florid complexion, thinks this is a sign of health. But presently, in middle life, and while still apparently strong and vigorous, So-and-So dies, much to the surprise of his friends, who never guessed that these additional stones meant a load of waste matter which the tired organs could not eliminate. The man who over-eats does not always get the kindly warning of a severe illness; often enough he goes on unchecked until he dies twenty or thirty years before nature meant it. But just this sudden death prevents the trouble from being suspected, that years of over-eating have been rendering him less able to withstand some accidental exposure, some unwonted exertion, some unusual strain. Dr. Keith's prescription, then, is: Moderation at all times; in illness, complete or nearly complete fasting. Do not administer drugs to force nature to go on with her work, but wait until she gathers strength in her own way.

This is the burden of Dr. Keith's book, and though it is his mission in this specially to denounce excesses in food, we should do him an injustice if we left it to be inferred that he approves of alcohol. In practice we should expect to find him most unwilling to prescribe anything of the sort. Not only does he point out that alcohol lowers the chemical activities of the body, and that its value as a stimulant is so evanescent that to be effective a fresh dose would have to be administered every half-hour, which would be manifestly injudicious, but he is of opinion that old people can endure alcohol less well than the young, their powers of elimination being weaker. To many people this will be an entirely new view of the matter, but as a matter of fact we find everywhere old people who instinctively have given up wine with advancing age, feeling that it did not agree with them. Dr. Keith, moreover, would scarcely permit us the luxury of false teeth. When the teeth fail us it is a sign that the food which requires their aid for mastication should be given up also, and we should reduce ourselves to a diet similar to that of our toothless infant day.

Such is Dr. Keith's evangel. While there are points in it which all could not endorse, it is certainly a doctrine which all might do well to ponder in a sensuous and luxurious age.

